# Supplementary material for: Rapid termination of the African Humid Period triggered by northern high-latitude cooling
Source: Nat Commun. 2017 Nov 8;8:1372. doi: 10.1038/s41467-017-01454-y (PMC5678106; doi:10.1038/s41467-017-01454-y)
Supplement: Supplementary file 1 — Supplementary Information [file 41467_2017_1454_MOESM1_ESM.pdf]

Supplementary Note 1: Leaf-wax *n*-alkane δD

The C_{29} and C_{31} *n*-alkanes were the most abundant homologues in the GeoB4905-4 sediments. The C_{29} *n*-alkane is more dominantly produced by woody plants and forbs, which typically perform C_3 photosynthesis (*1*), in contrast to the C_{31} *n*-alkane, which is more evenly produced by trees (C_3) and grasses (C_4 plants in this area). The C_{29} and C_{31} *n*-alkane δD records display a similar evolution (**Supplementary Fig. 2**). δD_{31} values are on average 5‰ lower, likely due to the greater relative contribution of the C_{31} *n*-alkane from grasses (*2, 3*), which are thought to partially use unenriched xylem water during biosynthesis (*4*). Nonetheless, the similar δD evolution of the two homologues suggests a minor effect of any changes in vegetation source on temporal evolution of the climatic signal. The C_{29} *n*-alkane yielded more precise δD values than the C_{31} *n*-alkane, and because of its more restricted vegetation source may be less sensitive to vegetation change. Thus, we report δD values for the C_{29} *n*-alkane, denoted as δD_{wax}.

Supplementary Note 2: Leaf-wax $\delta^{13}C$ and vegetation type

The higher relative contribution of the C_{31} *n*-alkane from grasses likely explains the slightly higher $\delta^{13}C$ values of this homologue (**Supplementary Fig. 2**). The C_{29} *n*-alkane yielded similar but analytically more precise $\delta^{13}C$ values than the C_{31} homologue and so we report values for the C_{29} *n*-alkane, denoted as $\delta^{13}C_{wax}$. $\delta^{13}C_{wax}$ values were lowest between 25 ka and 18 ka, remained relatively stable from the Younger-Dryas (Y-D; 12.7 ka - 11.7 ka) into the AHP and increased from the mid to late Holocene (**Supplementary Fig. 2**). Given that the modern vegetation distribution is a function of mean annual precipitation and wet season length (e.g. *5*), it might be expected that this reflects hydrological changes. However, the $\delta^{13}C_{wax}$ evolution is at odds with the nearby Barombi Mbo pollen record (*6*), which displays a lower abundance of forest pollen during the last

glacial period, a marked increase during the AHP and a decrease during the late Holocene, likely reflecting precipitation changes, in line with lake levels (*7*). This hence suggests precipitation changes were not the cause of the first-order C_3-C_4 vegetation shifts recorded in Gulf of Guinea sediments. Rather, it is likely that $\delta^{13}C_{wax}$ from core GeoB4905-4 is reflecting small shifts in the relative contribution of C_3 dominated material from southern Cameroon versus C_4 dominated material from the Sahelian-Saharan region. In particular, we suggest that the lack of a deglacial C_3 increase in our record is mainly due to deglacial sea-level rise shifting the mouths of the Ntem, Nyong and Sanaga Rivers inland, reducing the C_3 contribution from these rivers relative to the Sahelian dust sources. The sea level change between the last glacial maximum and today is highlighted by comparison of the 120m isobath and the modern coastline (**Fig. 1b**). Sea level rise may have also opened up an additional transport pathway for Niger River material to reach the core site.

Supplementary Note 3: *i*LOVECLIM transient model simulation

We compared our δD_{wax} record with δD_p and annual precipitation amount from a transient run of the intermediate complexity isotope-enabled climate model *i*LOVECLIM (*8-11*). Concerning long-term trends, there is a reasonable resemblance between GeoB4905-4 δD_{wax} and modelled Cameroon δD_p (**Supplementary Fig. 4a,b**). The δD_{wax} exhibits larger magnitude changes than modelled δD_p, which may be due to the additional effect of relative humidity on the δD_{wax}. The model does not, however, reproduce the rapid transitions evident in the δD_{wax} record, likely because the model is of intermediate complexity and an accelerated forcing technique was used (*81*). Freshwater fluxes induced by ice-sheet collapses are not included, likely explaining the absence of rapid changes at the HS1 and YD terminations, and relevant vegetation, soil moisture and dust feedbacks are likely not adequately accounted for in the model, thus excluding their potential role in the enhancement of aridification at the AHP

termination. Despite these potential shortcomings, the *i*LOVECLIM model shows that both Cameroon δD_p and Sahel-Sahara δD_p display a similar evolution to Sahel precipitation amount, but a different evolution to Cameroon precipitation amount (**Supplementary Fig. 4b-e**). This would suggest that Sahelian-Saharan precipitation amount exerts a control on Cameroon δD_p.

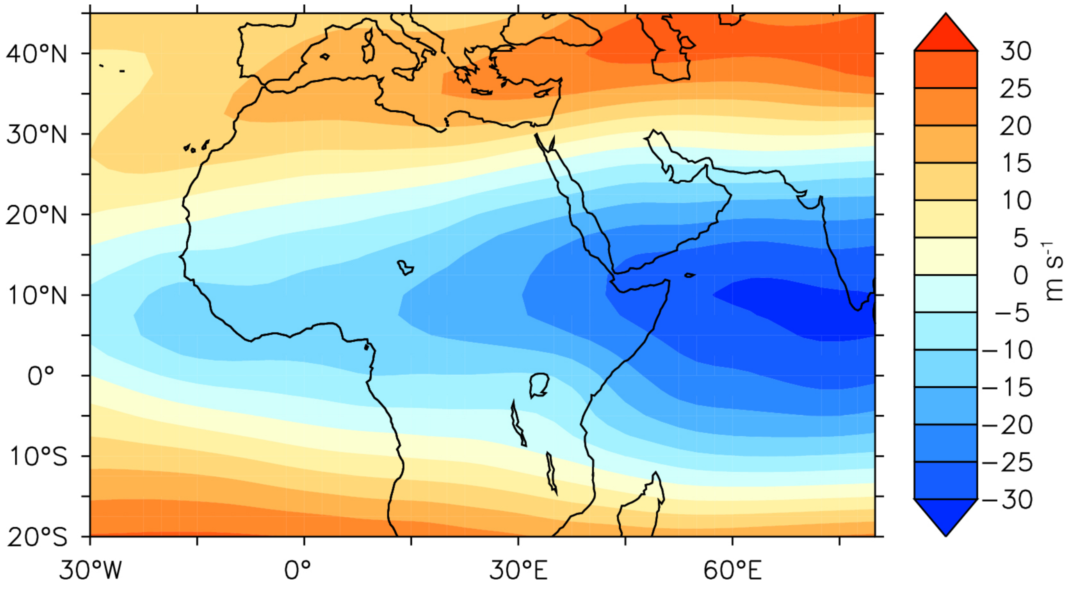

Supplementary Figure 1. Tropical Easterly Jet. Map of zonal windspeed (m s^{-1}) in the upper troposphere (150 hPa) during boreal summer (JJA) from NCEP re-analysis data (*12*); climatological mean. Negative values (blue colors) represent easterly winds.

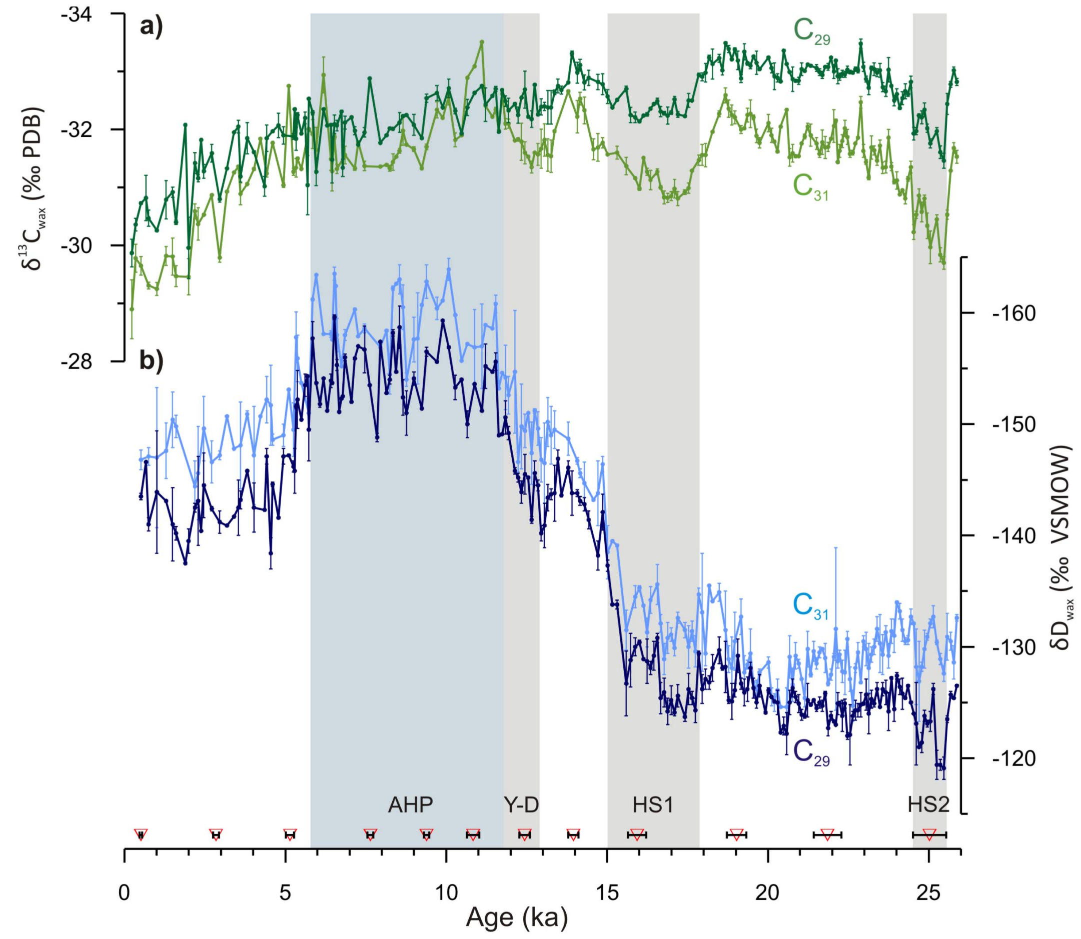

Supplementary Figure 2. Raw $\delta^{13}C_{wax}$ and δD_{wax} data. a) $\delta^{13}C_{wax}$ of the *n*-C_{29} (dark green) and *n*-C_{31} (light green) homologues from core GeoB4905-4. Error bars represent analytical precision. **b)** δD_{wax} of the *n*-C_{29} (dark blue) and *n*-C_{31} (light blue) homologues from GeoB4905-4. Error bars represent analytical precision. Vertical bars mark African Humid Period (AHP), Younger-Dryas (Y-D) and Heinrich Stadials 1 and 2 (HS1 and HS1).

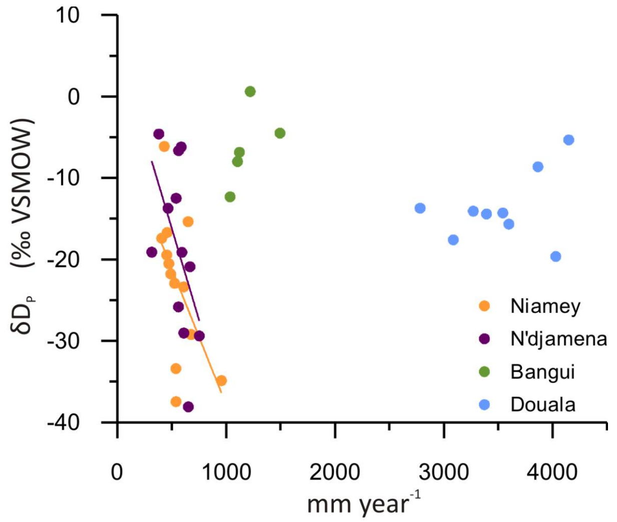

Supplementary Figure 3. Precipitation amount effect in the Sahel and Cameroon. Precipitation-weighted annual-mean δD_p and annual-mean precipitation amount for Sahelian (Niamey, Niger and N'djamena, Chad) and equatorial (Douala, southern Cameroon and Bangui, Central African Republic) GNIP stations closest to the core site (*13*). δD_p is negatively correlated with local precipitation amount at Niamey ($r = -0.56$, $p = 0.024$) and N'djamena ($r = -0.52$, $p = 0.042$), but there is no significant correlation at Douala and Bangui. The lack of correlation may partly reflect lack of data. Nonetheless, model data and the OIPC interpolated data from West Africa show a less steep relationship between δD_p and precipitation amount in wetter regions (*14*).

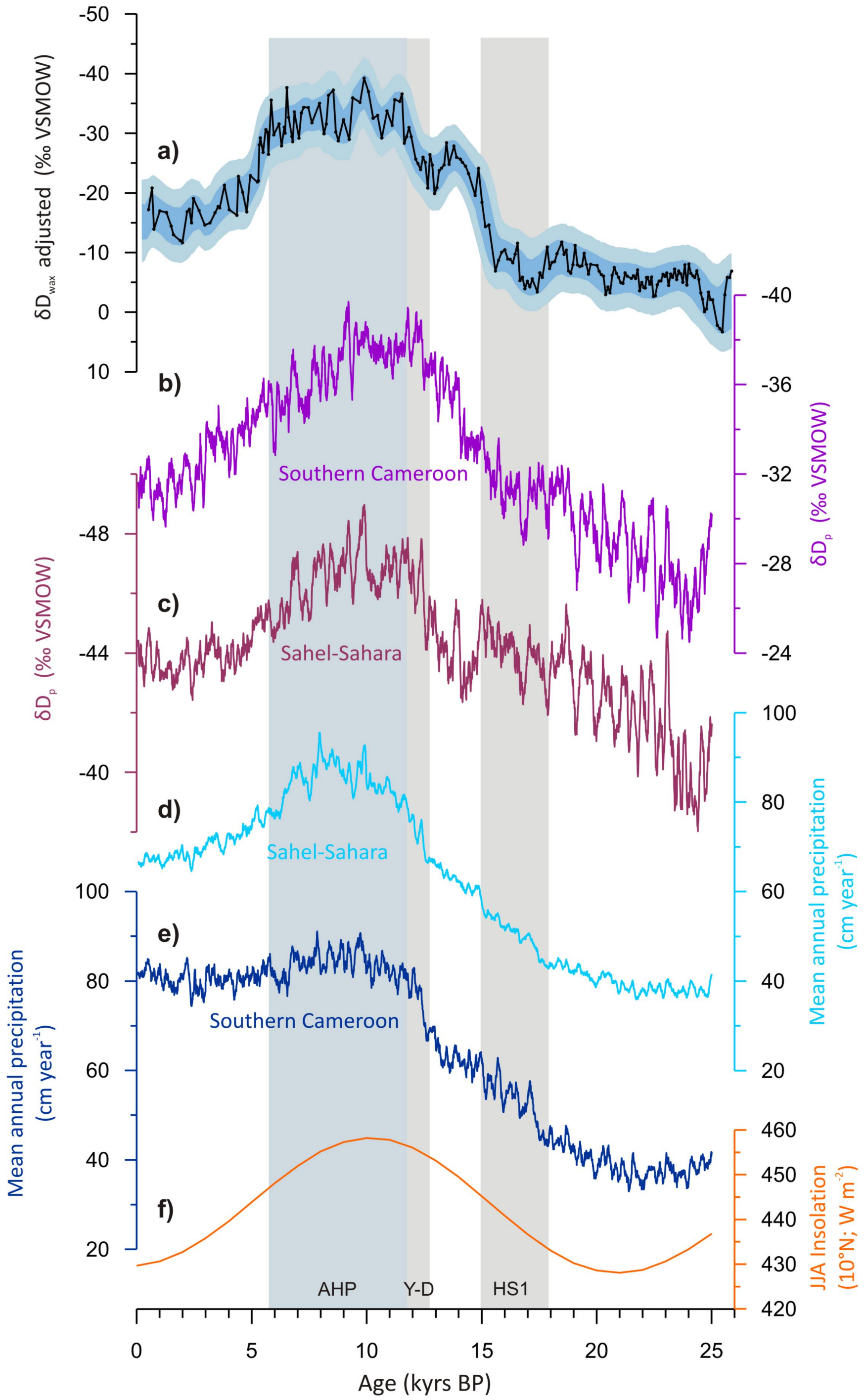

Supplementary Figure 4. Comparison of GeoB4905-4 δD$_{wax}$ with *i*LOVECLIM δD$_p$ and precipitation amount for the Sahel-Sahara and southern Cameroon. a) δD$_{wax}$ (ice-volume and vegetation adjusted). **b)** *i*LOVECLIM southern Cameroon δD$_p$. **c)** *i*LOVECLIM Sahel-Sahara δD$_p$. **d)** *i*LOVECLIM Sahel-Sahara precipitation amount, which correlates well with Sahel-Sahara δD$_p$ and southern Cameroon δD$_p$. **e)** *i*LOVECLIM

southern Cameroon precipitation amount. **f)** Insolation for JJA at 10°N (*15*). The southern Cameroon region is defined as (9°E-14°E and 1°N-6°N) and the Sahel-Sahara as (0°E-25°E, 10°N-20°N).

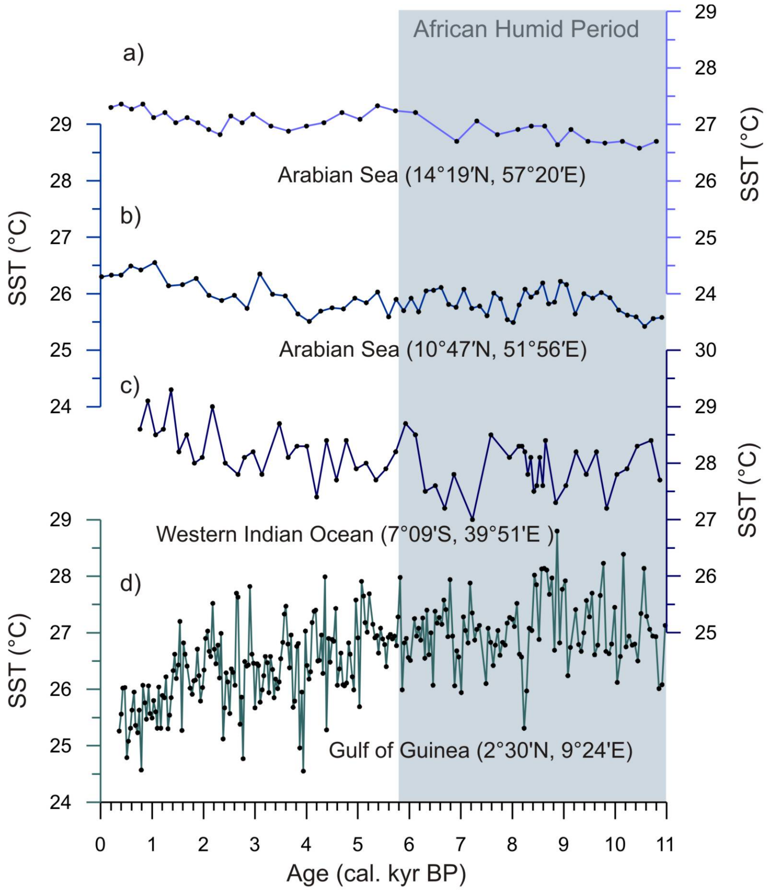

Supplementary Figure 5. Tropical Sea Surface Temperatures. a) Arabian Sea surface temperature (SST) based on alkenone $U_{37}^{K'}$ from core SO42-74KL (*16*). **b)** Arabian Sea SST based on alkenone $U_{37}^{K'}$ from core NIOP-C2-905 (*16*). **c)** Western Indian Ocean SST based on foraminiferal Mg/Ca from core GeoB12615-4 (*17*). **d)** Gulf of Guinea SST based on foraminiferal Mg/Ca from core MD03-2707 (*22*). These records do not show a significant decrease at 5.5 ka below the threshold for deep convection, estimated to be 26-28°C (*18*). The blue box represents the African Humid Period (AHP).

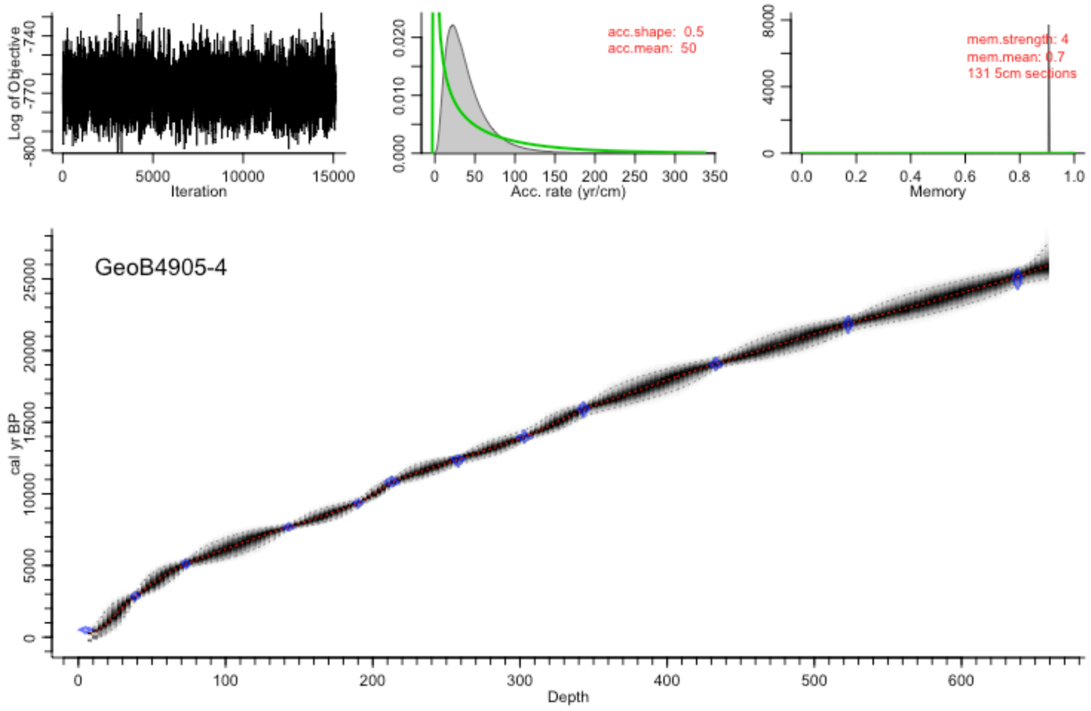

Supplementary Figure 6. Age-depth model for marine sediment core GeoB4905-4 produced using BACON2.2. Blue symbols indicate individual calibrated radiocarbon ages, grey shading indicates all likely age depth models, black dotted lines the 95% confidence intervals, and the red dotted line the median age-depth model.

Location / Core	Proxy	Temperature Calibration Reference	Latitude (°)	Longitude (°)	Elevation (m.a.s.l.)	Published Seasonal Interpretation	Reference	PC 1 Loading
MD95-2043	UK'37	(*19*)	36.1	-2.6	-1000	Annual	(*20*)	0.30
M39-008	UK'37	(*19*)	36.4	-7.1	-576	Annual	(*20*)	0.34
MD95-2011	UK'37	(*19*)	67	7.6	-1048	Summer	(*20*)	0.41
GeoB5901-2	UK'37	(*19*)	36.4	-7.1	-574	Annual	(*21*)	0.34
MD95-2015	UK'37	(*19*)	58.8	-26	-2630	Annual	(*22*)	0.36
IOW225517	UK'37	(*19*)	57.7	7.1	-293	Summer	(*23*)	0.44
JR51GC-35	UK'37	(*19*)	67	-18	-420	Annual	(*24*)	0.16
D13822	UK'37	(*19*)	38.6	-9.5	-88	Summer	(*25*)	0.40

Supplementary Table 1. Northeast Atlantic SST records used in the Empirical Orthogonal Function (EOF) analysis, following a previous approach (*26*). For consistency with our model simulations, we selected only northeast Atlantic records from a previous Holocene temperature compilation (*27*). To minimize seasonal bias we selected only alkenone SST records, commonly interpreted as reflecting summer or annual temperature. Age models are re-calibrated with the Marine09 calibration, as previously (*27*). Before EOF analysis, records were re-sampled every 0.1 kyr between 1.8 and 8.4 ka and normalized to the standard deviation of this period.

Supplementary References

1. J. D. Hemingway, E. Schefuß, B. J. Dinga, H. Pryer, V. V. Galy, Multiple plant-wax compounds record differential sources and ecosystem structure in large river catchments. *Geochim. Cosmochim. Acta* **184**, 20-40 (2016).

2. A. Vogts, H. Moossen, F. Rommerskirchen, J. Rullkötter, Distribution patterns and stable carbon isotopic composition of alkanes and alkan-1-ols from plant waxes of African rain forest and savanna C_3 species. *Org. Geochem.* **40**, 1037-1054 (2009).

3. F. Rommerskirchen, A. Plader, G. Eglinton, Y. Chikaraishi, J. Rullkötter, Chemotaxonomic significance of distribution and stable carbon isotopic composition of long-chain alkanes and alkan-1-ols in C_4 grass waxes. *Org. Geochem.* **37**, 1303-1332 (2006).

4. A. Kahmen, E. Schefuß, D. Sachse, Leaf water deuterium enrichment shapes leaf wax *n*-alkane δD values of angiosperm plants I: Experimental evidence and mechanistic insights. *Geochim. Cosmochim. Acta* **111**, 39-49 (2013); 10.1016/j.gca.2012.09.003.

5. J. A. Collins, E. Schefuß, D. Heslop, S. Mulitza, M. Prange, M. Zabel, R. Tjallingii, T. M. Dokken, E. Huang, A. Mackensen, M. Schulz, J. Tian, M. Zarriess, G. Wefer, Interhemispheric symmetry of the tropical African rainbelt over the past 23,000 years. *Nat. Geosci.* **4**, 42-45 (2011).

6. J. Maley, P. Brenac, Vegetation dynamics, palaeoenvironments and climatic changes in the forests of western Cameroon during the last 28,000 years BP. *Rev. Palaeobot. Palynol.* **99**, 157-187 (1998).

7. F. Gasse, Hydrological changes in the African tropics since the Last Glacial Maximum. *Quat. Sci. Rev.* **19**, 189-211 (2000).

8. D. Roche, δ^{18}O water isotope in the *i*LOVECLIM model (version 1.0)–Part 1: Implementation and verification. *Geoscientific Model Development* **6**, 1481-1491 (2013).

9. T. Caley, D. Roche, δ^{18}O water isotope in the *i*LOVECLIM model (version 1.0)–Part 2: Evaluation of model results against observed δ^{18}O in water samples. *Geoscientific Model Development* **6**, 1493-1504 (2013).

10. T. Caley, D. Roche, δ^{18}O water isotope in the *i*LOVECLIM model (version 1.0)–Part 3: A palaeo-perspective based on present-day data–model comparison for oxygen stable isotopes in carbonates. *Geoscientific Model Development* **6**, 1505-1516 (2013).

11. T. Caley, D. M. Roche, H. Renssen, Orbital Asian summer monsoon dynamics revealed using an isotope-enabled global climate model. *Nature communications* **5**, (2014); 10.1038/ncomms6371.

12. E. Kalnay, M. Kanamitsu, R. Kistler, W. Collins, D. Deaven, L. Gandin, M. Iredell, S. Saha, G. White, J. Woollen, The NCEP/NCAR 40-year reanalysis project. *Bull. Am. Meteorol. Soc.* **77**, 437-471 (1996).

13. IAEA/WMO, Global Network of Isotopes in Precipitation. The GNIP Database. Accessible at: http://www.iaea.org/water. (2006).

14. J. E. Tierney, F. S. Pausata, P. deMenocal, Rainfall regimes of the Green Sahara. *Science advances* **3**, e1601503 (2017).

15. A. Berger, Long-term variations of caloric insolation resulting from the earth's orbital elements. *Quaternary Research* **9**, 139-167 (1978).

16. C. Huguet, J.-H. Kim, J. S. Sinninghe Damsté, S. Schouten, Reconstruction of sea surface temperature variations in the Arabian Sea over the last 23 kyr using organic proxies (TEX_{86} and $U^{K'}_{37}$). *Paleoceanography* **21**, (2006); 10.1029/2005PA001215.

17. S. Romahn, A. Mackensen, J. Groeneveld, J. Pätzold, Deglacial intermediate water reorganization: new evidence from the Indian Ocean. *Clim. Past* **10**, 293-303 (2014).

18. C. Zhang, Large-scale variability of atmospheric deep convection in relation to sea surface temperature in the tropics. *J. Clim* **6**, 1898-1913 (1993).

19. P. J. Müller, G. Kirst, G. Ruhland, I. Von Storch, A. Rosell-Melé, Calibration of the alkenone paleotemperature index $U^{K'}_{37}$ based on core-tops from the eastern South Atlantic and the global ocean (60° N-60° S). *Geochim. Cosmochim. Acta* **62**, 1757-1772 (1998).

20. I. Cacho, J. O. Grimalt, M. Canals, L. Sbaffi, N. J. Shackleton, J. Schönfeld, R. Zahn, Variability of the western Mediterranean Sea surface temperature during the last 25,000 years and its connection with the Northern Hemisphere climatic changes. *Paleoceanography* **16**, 40-52 (2001).

21. J.-H. Kim, N. Rimbu, S. J. Lorenz, G. Lohmann, S.-I. Nam, S. Schouten, C. Rühlemann, R. R. Schneider, North Pacific and North Atlantic sea-surface temperature variability during the Holocene. *Quat. Sci. Rev.* **23**, 2141-2154 (2004).

22. O. Marchal, I. Cacho, T. F. Stocker, J. O. Grimalt, E. Calvo, B. Martrat, N. Shackleton, M. Vautravers, E. Cortijo, S. van Kreveld, Apparent long-term cooling of the sea surface in the northeast Atlantic and Mediterranean during the Holocene. *Quat. Sci. Rev.* **21**, 455-483 (2002).

23. K.-C. Emeis, U. Struck, T. Blanz, A. Kohly, M. Voβ, Salinity changes in the central Baltic Sea (NW Europe) over the last 10000 years. *The Holocene* **13**, 411-421 (2003).

24. J. A. Bendle, A. Rosell-Melé, High-resolution alkenone sea surface temperature variability on the North Icelandic Shelf: implications for Nordic

Seas palaeoclimatic development during the Holocene. *The Holocene* **17**, 9-24 (2007).

25. T. Rodrigues, J. O. Grimalt, F. G. Abrantes, J. A. Flores, S. M. Lebreiro, Holocene interdependences of changes in sea surface temperature, productivity, and fluvial inputs in the Iberian continental shelf (Tagus mud patch). *Geochemistry, Geophysics, and Geosystems* **10**, (2009).

26. J.-H. Kim, H. Meggers, N. Rimbu, G. Lohmann, T. Freudenthal, P. J. Müller, R. R. Schneider, Impacts of the North Atlantic gyre circulation on Holocene climate off northwest Africa. *Geology* **35**, 387-390 (2007).

27. S. A. Marcott, J. D. Shakun, P. U. Clark, A. C. Mix, A reconstruction of regional and global temperature for the past 11,300 years. *Science* **339**, 1198-1201 (2013).
